# A Valuable and Low-Budget Process Scheme of Equivalized 1 nm Technology Node Based on 2D Materials

**DOI:** 10.1007/s40820-025-01702-7

**Published:** 2025-03-18

**Authors:** Yang Shen, Zhejia Zhang, Zhujun Yao, Mengge Jin, Jintian Gao, Yuhan Zhao, Wenzhong Bao, Yabin Sun, He Tian

**Affiliations:** 1 College of Integrated Circuit Science and Engineering, Shanghai Key Laboratory of Multidimensional Information Processing, East China Normal University, Shanghai, 200241 People’s Republic of China; 2 Institute of Microelectronics and Beijing National Research Center for Information Science and Technology (BNRist), Tsinghua University, Beijing, 100084 People’s Republic of China; 3 State Key Laboratory of ASIC and System, School of Microelectronics, Fudan University, Shanghai, 200433 People’s Republic of China; 4 Shaoxin Laboratory, Shaoxing, 312000 People’s Republic of China

**Keywords:** Two-dimensional semiconductors, 1 nm technology node, Nanosheet field-effect transistors, Complementary field-effect transistors, Horizontal scaling

## Abstract

Emerging two-dimensional (2D) semiconductors are among the most promising materials for ultra-scaled transistors due to their intrinsic atomic-level thickness. As the stacking process advances, the complexity and cost of nanosheet field-effect transistors (NSFETs) and complementary FET (CFET) continue to rise. The 1 nm technology node is going to be based on Si-CFET process according to international roadmap for devices and systems (IRDS) (2022, https://irds.ieee.org/), but not publicly confirmed, indicating that more possibilities still exist. The miniaturization advantage of 2D semiconductors motivates us to explore their potential for reducing process costs while matching the performance of next-generation nodes in terms of area, power consumption and speed. In this study, a comprehensive framework is built. A set of MoS_2_ NSFETs were designed and fabricated to extract the key parameters and performances. And then for benchmarking, the sizes of 2D-NSFET are scaled to a extent that both of the Si-CFET and 2D-NSFET have the same average device footprint. Under these conditions, the frequency of ultra-scaled 2D-NSFET is found to improve by 36% at a fixed power consumption. This work verifies the feasibility of replacing silicon-based CFETs of 1 nm node with 2D-NSFETs and proposes a 2D technology solution for 1 nm nodes, i.e., “2D eq 1 nm” nodes. At the same time, thanks to the lower characteristic length of 2D semiconductors, the miniaturized 2D-NSFET achieves a 28% frequency increase at a fixed power consumption. Further, developing a standard cell library, these devices obtain a similar trend in 16-bit RISC-V CPUs. This work quantifies and highlights the advantages of 2D semiconductors in advanced nodes, offering new possibilities for the application of 2D semiconductors in high-speed and low-power integrated circuits.

**Supplementary Information:**

The online version contains supplementary material available at 10.1007/s40820-025-01702-7.

## Highlights

- A set of MoS_2_ nanosheet field-effect transistors (NSFETs) are fabricated, with two stacking nanosheet channel, in which two parallel MoS_2_ channels are controlled by three gate electrodes simultaneously.
- By building a comprehensive framework, the feasibility of replacing silicon-based complementary field-effect transistors of 1 nm node with 2D-NSFETs provides a 2D technology solution for 1 nm nodes, i.e., “2D eq 1 nm” nodes are verified.
- The horizontally miniaturized 2D-NSFET achieves a frequency increase of 28% at a fixed power consumption and also obtains a similar trend in 16-bit RISC-V CPU.

**Supplementary Information:**

The online version contains supplementary material available at 10.1007/s40820-025-01702-7.

## Graphical abstract

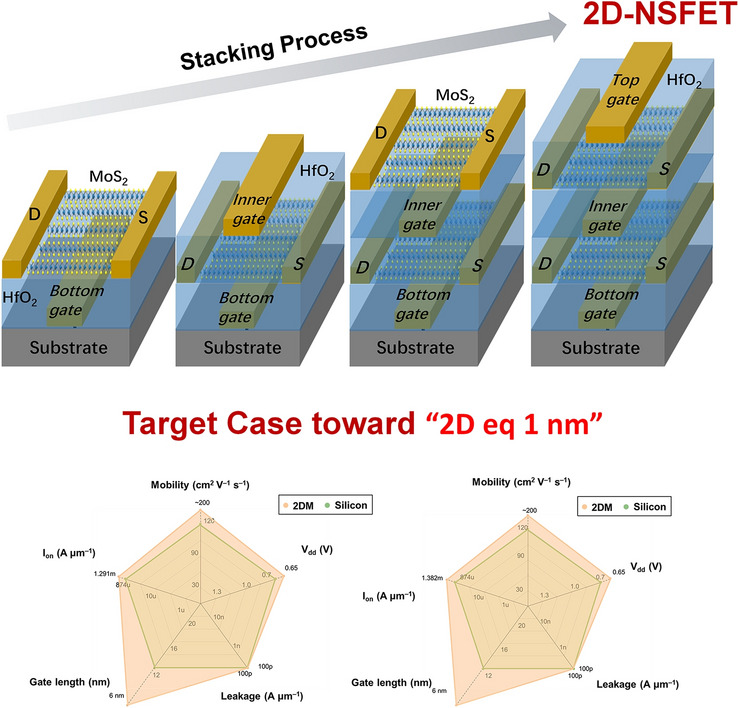

## Introduction

**Fig. 1:**
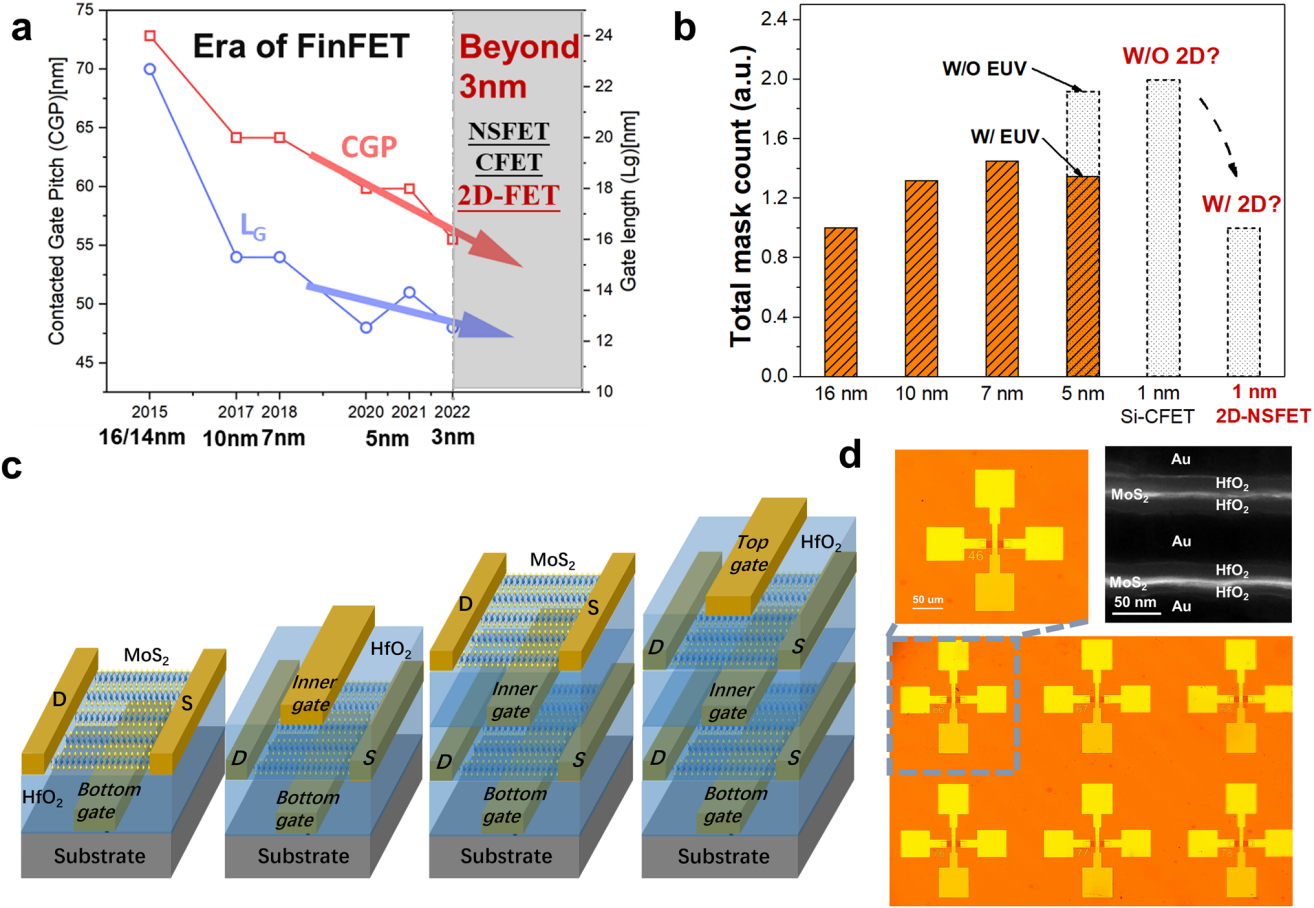
**a** Evolvement of gate length and CGP from 16 nm to the nodes beyond 3 nm; **b** Evolvement of total mask number from 16 to 5 nm node, in which the advent of EUV at 5 nm node realizes the reduction of mask number, and it is envisioned that at the 1 nm node, the use of 2D-NSFETs can realize the halving of photomask count compared to the Si-CFET at 1 nm node. The 1 nm node here is referenced from Si-based integrated circuits. **c** MoS_2_ NSFET process flow diagram with two layers of conducting channels; **d** Optical photographs of devices and arrays of MoS_2_ NSFETs, as well as cross-sectional STEM view of a typical MoS_2_ NSFET showing the distribution of 2D channels, gate dielectrics and gate metals

Semiconductor technology continues to evolve with silicon as the primary material. However, as feature sizes continue to shrink, traditional silicon-based field-effect transistors (FETs) are unable to maintain reliable and robust performance when channel lengths are reduced to less than 12 nm, due to short-channel effects (SCEs). As integration technology evolves to the 5 nm process node, increasing SCEs, reduced fin counts, weakened electrostatic control and increased fabrication complexity limit the miniaturization performance of FinFET devices [2–4]. In order to improve gate control for ultra-short channels and to ensure that sufficiently strong electrostatic control is constructed to meet the requirements of more advanced semiconductor processes, gate-all-around FETs (GAAFETs) have become the dominant device at the 5 nm and sub-5 nm nodes [5–7]. Nanosheet field-effect transistors (NSFETs) with the GAA structure have gained wide acceptance in the semiconductor industry for their excellent low power performance, enhanced ability to suppress SCEs, superior drive capability, lower parasitic capacitance, flexible channel size tunability and higher device reliability [8, 9]. Further, IMEC proposes new device structures such as Forksheet FET [10] and complementary FET (CFET) [11]. Due to their significant advantages in terms of power consumption, performance and area (PPA), CFETs have quickly emerged as a strong candidate for overcoming the forthcoming limitations in semiconductor device miniaturization and hold promise for applications beyond the 3 nm technology node (Fig. 1a). As the size continues to shrink, the underlying physical limitations that are difficult to completely resolve have prompted researchers to continue to explore other options.

Two-dimensional (2D) materials with their excellent semiconductor properties offer a new way to move forward [12]. One of the major challenges in downsizing the mainstream Si-FETs is the tunneling current between source and drain, especially when the channel length is shortened to below 20 nm where the off-state current (*I*_OFF_) cannot be suppressed. Taking MoS_2_ as an example, the wider bandgap (~ 2 eV) and higher effective mass (~ 0.55 m_0_) could help constrain the I_OFF_ and improve the switching ratio *I*_ON_/*I*_OFF_. In addition, the atomic-scale thickness gives MoS_2_ FETs excellent gate tuning properties. In recent years, 2D-FETs with 1 nm gate length (*L*_G_) and sub-1 nm *L*_G_ have been fabricated [13–15]. In contrast, the gate-length miniaturization of Si-FETs has gradually stagnated at 12 nm, and thus, 2D semiconductors represent a great opportunity if they can be optimized to exhibit acceptable performance.

Analyzing 2D transistors based on physical models can effectively predict the performance potential of new material transistors with small sizes at the device level. However, to evaluate the performance of 2D transistors in circuits, it is necessary to combine both physical models and circuit models, such as SPICE simulation. Although many theoretical analyses of 2D transistors have been carried out in academia at the device and circuit levels, few theoretical research has been reported on 2D transistors at advanced nodes. 2020, IMEC for the first time introduced DTCO, the Circuit-Device Process Co-optimization, to 2D devices [16]. Based on the Forksheet process, a dual-gate WS_2_ transistor was compared to a conventional Si-FETs at the 3 nm node using a calibrated BSIM-IMG model. By building a ring oscillation circuit, for example, power consumption and delay are extracted. The optimization of physical and structural parameters resulted in a 42% speedup compared to the Si-based 2 nm node. Meanwhile, by continuing to shorten the *L*_G_ and contacted gate pitch (CGP) of the WS_2_ Forksheet device to 8 and 36 nm, the speed can be further improved by 20% with an 8% area gain. In 2022, Lain-Jong Li et al. reported the performance evaluation of top gate and nanosheet transistors with MoS_2_ and WSe_2_ channels for SRAM circuits [17]. The results of device physics simulations show that the single-layer 2D-FETs can obtain the same level of I_ON_ as that of silicon when the contact resistance, equivalent oxide thickness EOT and mobility are optimized to 0.13 kΩ-μm, 0.5 nm and 354 cm^2^/(V-s), respectively. Compared to Si-FETs, the optimized 2D-FETs can reduce SRAM read time and write time by about 41% and 38%, respectively. Further, the work reported in 2024 compares the performance of 2D and Si-based SRAM circuits from 16 to 1 nm nodes in device architectures such as FinFETs and NS-GAAFETs [18].

Currently, there exist process challenges in 2D-NSFETs [19, 20]. How to prepare good electrical contacts and GAA structures is a problem to be solved in the future. Previous research works concentrated on 2D-FETs at advanced nodes have obtained performance gains on circuits by optimizing device parameters at the same nodes and quantified the targeted case in terms of contact, mobility, etc., which is suggested to be achieved [16, 21]. However, the role of 2D-FETs at the undetermined “1 nm” node remains unexplored. In other words, whether 2D semiconductors have their own technology scheme at 1 nm node, i.e., “2D eq 1 nm” node? Moreover, more possibilities of 2D-FETs at advanced nodes need to be further explored. For example, according to the excellent properties of 2D semiconductors, it is worthy to figure out whether it is possible to evaluate 2D-FETs and Si-FETs at different nodes with different processes and compare their behaviors. In other words, whether it is possible to use 2D-based circuits at previous generation of nodes to achieve circuit performance comparable to Si-based circuits at next-generation nodes with more complicated device process? Since advanced nodes imply more complex process steps and higher preparation costs, such an assumption implies that 2D-FETs can improve performance while also reducing cost.

NSFET indeed has shown certain advantages in suppressing short-channel effects and improving device performance. In comparison, CFET has significant advantages in terms of PPA, which makes it a strong candidate for the 1 nm node. Although our work focuses on the “2D eq 1 nm” scheme based on 2D-NSFETs, we also recognize the importance of considering other device structures. Regarding nanowire GAA transistors, they also have their own characteristics. Nanowire GAA transistors can provide better electrostatic control due to their three-dimensional structure. However, they also face challenges such as process complexity and difficulty in precisely controlling the nanowire diameter and pitch. In our study, we chose to explore the potential of 2D-NSFETs because of the unique atomic-scale thickness and excellent semiconductor properties of 2D materials, which offer new possibilities for device miniaturization.

In this work, we develop a cross-scale simulation framework that combines underlying material parameter calculations, technology computer-aided design device simulation, circuit simulation based on SPICE model and finally a system performance evaluation platform. This framework can be used to investigate the advantages of 2D-FETs at the circuit and system level, providing a relatively accurate benchmark. After constructing a RO circuit and a 16-bit CPU consisting of 9 different logic gates, we compare the power consumption and speed of 3 nm node (based on Si-NSFET process), conventional 1 nm node (based on the CFET process) and our proposed “2D eq 1 nm” node (based on the 2D-NSFET process). Our results show that 2D-NSFETs have excellent horizontal scalability, allowing the “2D eq 1 nm” to exceed the power and speed of the Si-CFET at 1 nm nodes, while their area is comparable.

## Design and Considerations

As the process nodes continue to iterate, the total photomask count, a significant indicator of manufacturing complexity, increases. At the 5 nm node, the introduction of EUV has led to a significant reduction in mask count. However, with the advent of 3D CFET process at 1 nm node, the total photomask count would possibly double on the original single device process. Here, we make an assumption that in the development of nodes with 2D semiconductors as channel materials, it is possible to avoid adopting the 3D stacking process at the stage of 1 nm node and instead carry on the lateral miniaturization based on the NSFET process, which is utilized at 3 nm node. To achieve that, it is necessary to ensure that the horizontally scaled 2D-NSFETs exhibit PPAs not weaker than that of the Si-based CFETs process at 1 nm node. In this case, the adoption of 2D semiconductors at 1 nm node is going to bring about dramatic reduction in the total photomask count, as EUV technology does at 5 nm node, which is advantageous to cut down process complexity and cost (Fig. 1b). Notably, all technology nodes here are referenced from Si-based integrated circuits (ICs).

**Fig. 2:**
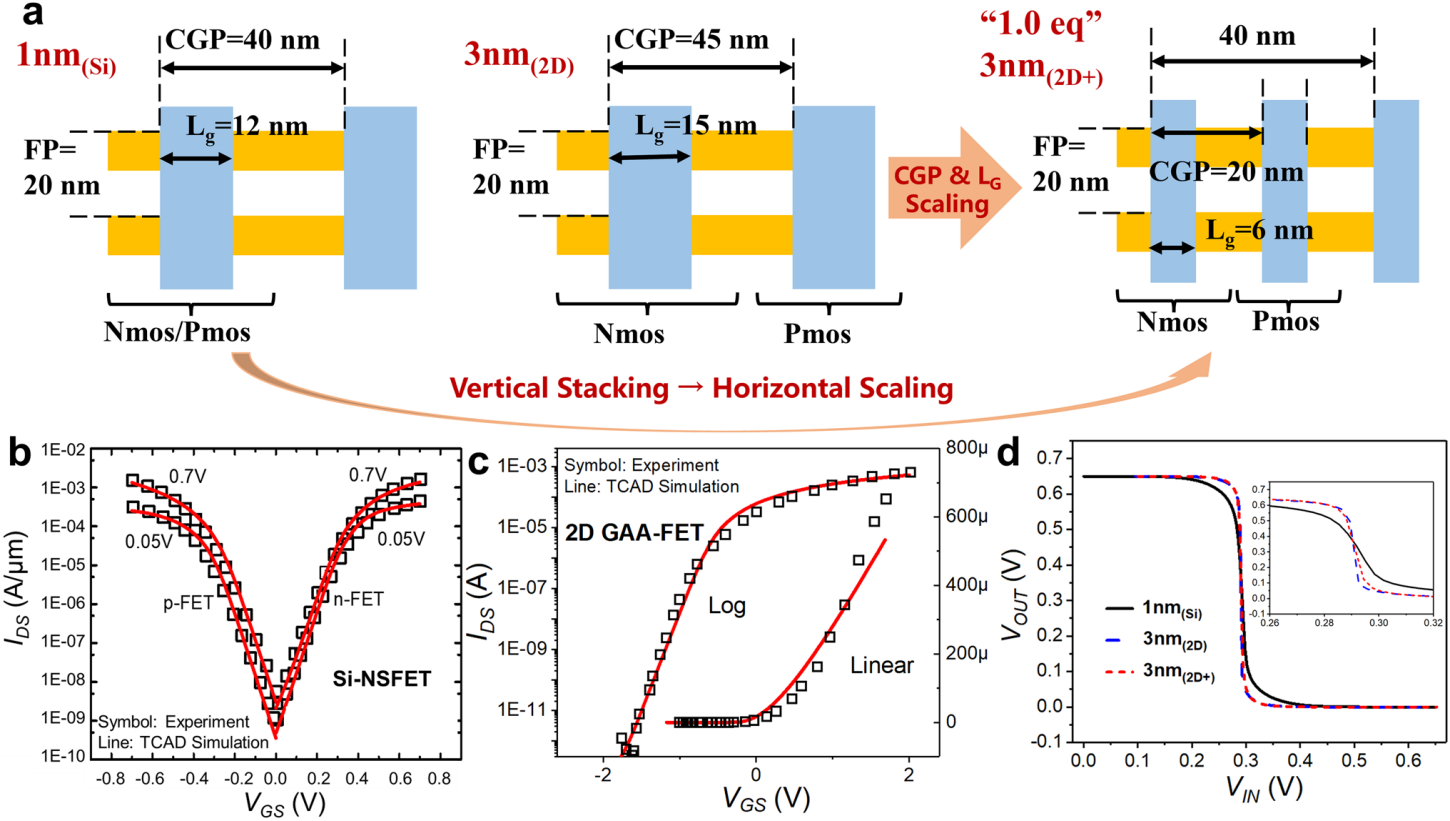
**a** Comparison of average device footprint for 1 nm_(Si)_, 3 nm_(2D)_ and 3 nm_(2D+)_ nodes; **b** Calibration of the simulated transfer characteristics to that of experimental Si-NSFET (*L*_G_ = 12 nm); **c** Calibration of the transfer characteristics of the 2D-NSFET; and **d** Voltage transfer characteristics (VTC) of the three different devices based inverter

To verify this idea, we first prepared a set of MoS_2_ NSFETs for calibrating our underlying physical model. Figure 1c shows a typical process and structural schematic of the 2D-NSFET prepared in this study. The channel material is high-quality 3-layer MoS_2_ film grown by chemical vapor deposition (CVD), in which two parallel MoS_2_ channels are controlled by three gate electrodes simultaneously. This design realizes a high-performance MoS_2_ NSFET with high mobility and low subthreshold swing, demonstrating the potential of this structure to improve device performance. A more detailed preparation flow is provided in Fig. S1. The MoS_2_ NSFET proposed in this study is promising for wafer-level large-scale integration. Figure 1d shows optical microscope images of MoS_2_ NSFETs and device arrays, confirming the high uniformity and scalability of the process. Figure 2d shows a cross section of a MoS_2_ NSFET device under high-resolution transmission electron microscope (STEM), from which the stacked structure of the 2-layer parallel MoS_2_ channel, HfO_2_ as the gate dielectric and the gate metal electrodes can be clearly recognized.

After electrical testing, Fig. S2 shows the transfer and output characteristic curves of five groups of MoS_2_ NSFETs. Under the test conditions of gate voltage *V*_GS_ = 3 V and drain voltage *V*_DS_ = 1 V, all five devices achieve an *I*_ON_ close to 100 μA, a threshold voltage *V*_th_ stabilized around -1 V and a switching ratio *I*_ON_/*I*_OFF_ of 10^9^. The behaviors indicate that the devices have excellent switching characteristics and current-driving capability. At *V*_GS_ = 3 V, the saturation current of the MoS_2_ NSFET averages over 200 μA, demonstrating good linear output characteristics. The results not only exhibit the excellent electrical performance of the MoS_2_ NSFET prepared in this work, but also demonstrate its significant advantages in terms of device homogeneity. This combination of homogeneity and high-performance shows that the process is promising for large-scale IC applications.

Although the above process still does not achieve a true match of device size at advanced nodes, we can make predictions based on the prepared devices. To benchmark 2D-NSFETs with Si-based CFETs at 1 nm nodes (the actual channel length is about 10 μm), the same area per device footprint should be ensured at first. For 1 nm node based on the Si-based CFET process (which can be referred to as 1 nm_(Si)_), the device gate pitch CGP is 40 nm and the *L*_G_ is 12 nm, according to IRDS. The CFET is a complementary stacked device, where the PMOS is stacked on top of the NMOS to halve the area per device footprint. For 3 nm node based on 2D-NSFET (called 3 nm_(2D)_), the CGP has to be halved to achieve the same footprint as the 1 nm_(Si)_ node. A scheme is shown in Fig. 2a, where the CGP and L_G_ are reduced to 20 and 6 nm, respectively, to halve the device footprint per unit device in planar dimension (called 3 nm_(2D+)_ or “2D eq 1 nm”). According to previous simulation results, 2D-NSFET maintains excellent characteristics at L_G_ of 6 nm. Therefore, this scheme essentially utilizes the lateral miniaturization capability of 2D-FETs as the equivalent of the Si-CFETs.

Figure S3 shows the structural parameters of the Si-FETs at 1 nm_(Si)_, 3 nm_(2D)_ and 3 nm_(2D+)_ nodes, according to which the corresponding device structures can be constructed, as shown in Fig. S4. For the simulation of Si-NSFETs at 3 and 1 nm node, the simulation includes many complex physical models at this scale. The details are described in Section Method. The electrical characteristics were simulated and calibrated with the experimental data reported in 2017 for NSFETs with 12 nm L_G_ (Fig. 2b). By setting the same structural dimensions and calibrating the physical parameters, the final experimental data and simulation can be in good agreement, which verifies the reasonableness of the above modeling setup. In order to more accurately simulate the performance of 2D-FETs, a cross-scale simulation framework is established, in which the underlying material parameters are calculated by density functional theory (DFT) and then brought into the device simulation for prediction (Fig. S5). In order to calibrate the MoS_2_ NSFET to the devices prepared above, the parameters calculated by DFT given in Fig. S6 are used and the *I*_DS_-*V*_GS_ curve is fitted as in Fig. 2c. In the device simulation, the multi-valley energy band parameters and 2D density of states model were introduced for the unique density of states distribution of 2D materials (Fig. S7).

In succession, WS_2_ NSFET is proposed to be used as the core device for 3 nm_(2D)_ node as well as 3 nm_(2D+)_ node. Compared with MoS_2_, WS_2_ has some unique electronic structure characteristics. Although MoS_2_ has been successfully fabricated into GAA transistors in our experiment and shows good performance, WS_2_ is more suitable for further miniaturization and optimization in the “2D eq 1 nm” scheme. In our simulation, with a mobility of 200 cm^2^/(V-s) (within the values realized so far) and a source-drain doping concentration of 1e20 cm^−3^, the *I*_ON_ of NMOS and PMOS corresponding to the 3 nm_(2D+)_ can reach 1.291 and 1.382 mA μm^−1^. More detailed parameter comparisons are presented in Fig. S8. Further, the calibrated BSIM-CMG model is obtained based on the device simulation data, and then, circuit simulation can be performed as shown in Fig. 2d. It is found that the devices at 3 nm_(2D+)_ and 3 nm_(2D+)_ node have higher inverter gain due to the better subthreshold characteristics of the 2D-FETs.

## Performance Comparison and Analysis

**Fig. 3:**
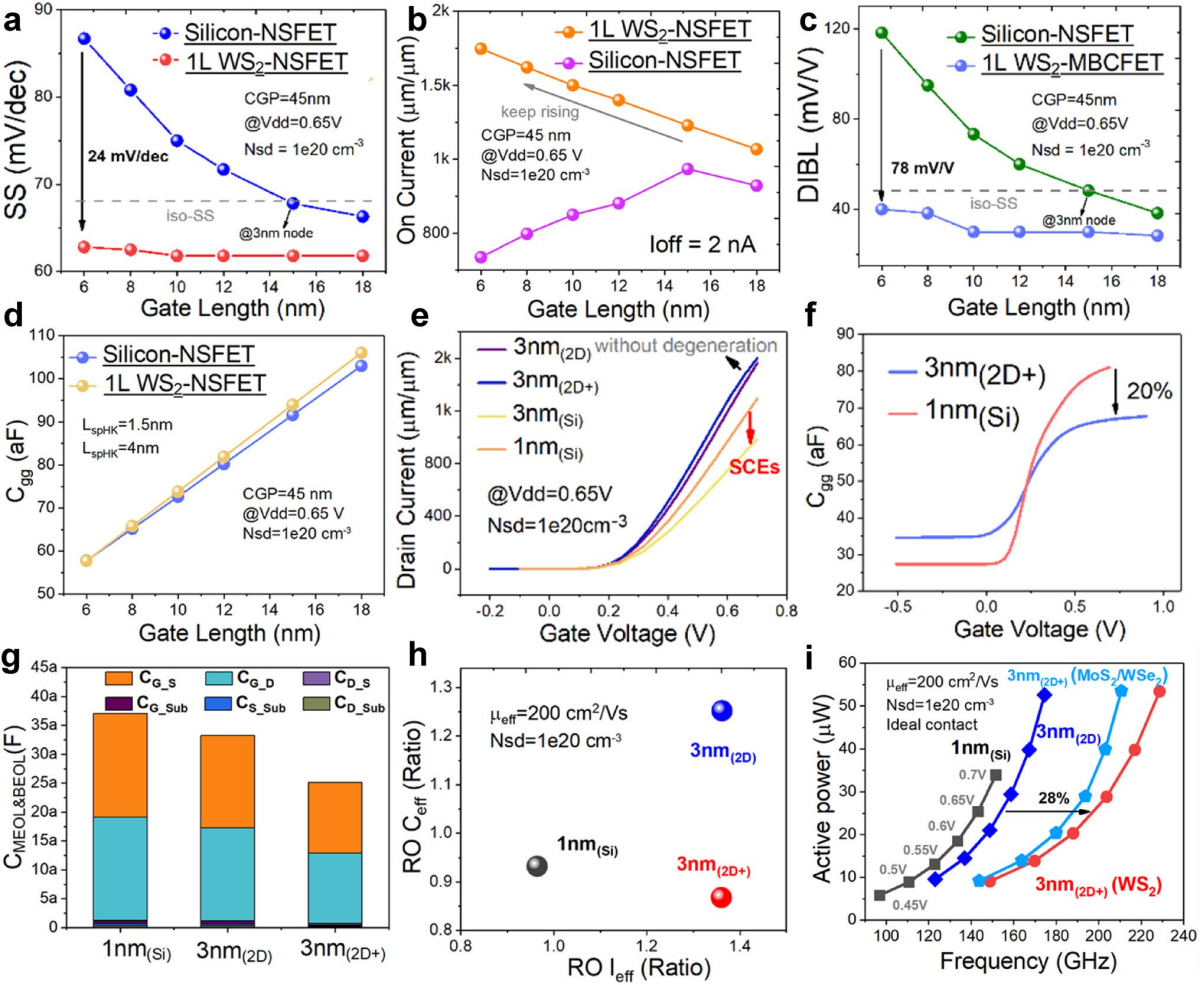
Comparison of electrical characteristics of devices at 3 nm_(2D+)_ node, 3 nm_(2D)_ node and 1 nm_(Si)_ node, with fixed CGP of 45 nm and *L*_G_ shrinking from 18 to 6 nm for Si-NSFETs and WS_2_ NSFETs. **a** SS variation, the SS of Si-NSFETs degrades drastically when the L_G_ is smaller than 12 nm, and **b** *I*_ON_ variation, thanks to the smaller feature length of 2D materials, the *I*_ON_ of WS_2_ NSFETs continues to increase even with the *L*_G_ scaled to 6 nm, while the I_ON_ of Si-NSFETs degrades continuously when the *L*_G_ is reduced to 6 nm; **c** DIBL variation, which follows a similar trend to that of SS; **d** C_gg_ variations, with EOT and gate size being the main influences on *C*_gg_; **e** Linear transfer characteristics corresponding to four devices, at 3 nm_(2D)_, 3 nm_(2D+)_, 3 nm_(Si)_ and 1 nm_(Si)_; **f** *C*_gg_-*V*_GS_ relationship for 3 nm_(2D+)_ and 1 nm_(Si)_ counterparts, which is reduced by 20% for the 3 nm_(2D+)_ due to shortened *L*_G_; **g** C_MEOL&BEOL_ comparison with middle end of line (MEOL) and back end of line (BEOL) parasitic capacitances; **h** Comparison of equivalent capacitances and equivalent currents extracted from RO circuits; and **i** Power–frequency comparison of RO circuits

The L_G_ is shortened from 18 to 6 nm to observe the performance change of the Si-NSFET and 2D-NSFET. Figure 3a–d shows the variation of SS, DIBL, *I*_ON_ and gate capacitance *C*_gg_ when *L*_G_ is varied. The supply voltage is fixed at 0.65 V, and the source-drain doping concentration *N*_sd_ is fixed at 1e20 cm^−3^. For the Si-NSFETs, when the *L*_G_ is smaller than 12 nm, serious short-channel effect occurs. Particularly, when the *L*_G_ is 6 nm, the SS is larger than 85 mV/dec^−1^ and the DIBL is close to 120 mV V^−1^. In contrast, using the 2D WS_2_ atomic channel, the SS and DIBL at 6 nm *L*_G_ are both smaller than the corresponding values of Si-NSFETs, with a decrease of 24 mV dec^−1^ and 78 mV V^−1^ at 6 nm *L*_G_, respectively. It indicates that 2D semiconductors have an excellent ability to scale the lateral dimensions and are able to operate at 6 nm *L*_G_ without significant short-channel effects. Therefore, benefiting from the decreasing channel length without degradation of SS, *I*_ON_ of WS_2_ NSFETs keeps increasing, contrary to degrading trend of *I*_ON_ of the silicon-based NSFETs. This shows that 2D-NSFETs are able to maintain the increasing driving capability when the *L*_G_ is smaller than 12 nm. The main reason is the degradation of SS and mobility of Si-NSFETs, while 2D materials can theoretically avoid the mobility degradation caused by channel thinning or other factors from the naturally occurring dangling bonds. Therefore, in the 2D-NSFET simulation, we set up a constant mobility model and weaken the corresponding mobility degradation model. The capacitance of the WS_2_ NSFETs remains at a similar level as that of the Si-NSFETs. Comparatively, the EOT and gate size are the main influencing factors of *C*_gg_, rather than the channel. This suggests that the main advantage of 2D-NSFETs is the ability to minimize the size, which can effectively increase the circuit speed by shortening the *L*_G_. The *I*_ON_ of the device actually affects the power consumption of the circuit, while the capacitance affects the speed of the circuit operation.

Using the above parameters, the electrical characteristics of devices corresponding to nodes such as 3 nm_(2D+)_, the 1 nm_(Si)_, 3 nm_(Si)_ based on NSFET and 3 nm_(2D)_ are simulated (Fig. 3e). The I_DS_ of the 3 nm_(2D+)_ node formed by the miniaturization of the 2D-NSFET has almost no degradation. In contrast, the Si-based device shows a slight increase in I_DS_ when going from 3 to 1 nm node, which corresponds to a shortening of the L_G_ from 15 to 12 nm. When the I_ON_ is degraded and the current at *V*_GS_ = *V*_DD_ is elevated, it means that the *I*_OFF_ is elevated and the device *I*_ON_/*I*_OFF_ is reduced, which leads to an increase in power consumption. This is a reflection of the short-channel effect in Si-based devices.

The *C-V* characterization of the devices at 1 nm_(Si)_ and 3 nm_(2D+)_ node is simulated, and the results are obtained as shown in Fig. 3f. The gate capacitance of the device at 3 nm_(2D+)_ node is reduced by about 20% compared to the 1 nm_(Si)_ due to the reduction of *L*_G_ to only 6 nm.

In order to demonstrate the variation of parasitic capacitance of each part inside the device, Fig. 3g shows the *C*_MEOL&BEOL_ capacitance between the four ports of the device, including *C*_G_S_, *C*_G_D_, *C*_D_S_, *C*_G_sub_, *C*_D_sub_ and *C*_S_sub_. The *C*_G_S_ and *C*_G_D_ are the main part of *C*_gg_, and they decrease with L_G_ decreasing, which is in accordance with the results in Fig. 3f. Meanwhile, Fig. S9 zooms in on the comparison of the other four capacitors. Compared to 1 nm_(Si)_ and 3 nm_(2D)_, 3 nm_(2D+)_ shows decrease in all four parts except for *C*_D_S_, which can be own to the decrease in the source–drain spacing.

The above results show that 3 nm_(2D+)_ has a theoretical advantage over 1 nm_(Si)_ in terms of both power consumption and speed. To quantify this advantage at the circuit level, a 15-step ring oscillator (RO) circuit is constructed with three fan-outs for each inverter stage. Figure 3h shows the equivalent capacitance and equivalent current extracted for the three devices mentioned above with a fixed voltage *V*_DD_ of 0.65 V. Similar to the trends of previously extracted *I*_ON_ and gate capacitance, the 3 nm_(2D+)_ node gives the smallest equivalent capacitance and largest equivalent current.

As the supply voltage of the RO is gradually increased from 0.45 to 0.7 V, the relationship between active power and frequency is extracted by circuit simulation for devices at different nodes, as shown in Fig. 3i. The 3 nm_(2D)_ node, that is, 3 nm nodes based on 2D-NSFET devices, outperforms the 1 nm_(Si)_ in terms of power consumption and speed. However, the area per device footprint of them is not in similar level. Not only the devices at 3 nm_(2D+)_ node achieve the same area efficiency, but also at the same time power consumption and area are significantly improved. At a fixed power, in terms of frequency, the 3 nm_(2D+)_ offers a 28% improvement over 3 nm_(2D+)_ and a 36% improvement over 1 nm_(Si)_.

## System-Level Demonstration

Currently reported MoS_2_ transistor-based CPUs mostly consist of up to 100 transistors, and the layout is drawn manually [22]. The drawback of manual layout drawing is that the scale of the system is limited, which is fine for 1-bit CPUs. But for large-scale circuits, it is too labor-intensive and difficult to ensure reliability. In addition, current logic gate circuits based on 2D semiconductors are using long channel two-dimensional devices with a large cell footprint, while short-channel 2D-based circuits are seldom reported. Achieving small-scale integration based on ultra-scaled 2D-FETs can help to truly improve chip integration and reduce the system footprint with flat power consumption and latency [23]. In 2023, hybrid 2D/CMOS-based microchips were reported to be designed using standard EDA tools, and the scale was further improved [24].

Although 2D integrated circuits are already available at some scale, however, the necessary bridge between laboratory-prepared 2D devices and final system applications is still lacking. To facilitate IMEC’s expectation that 2D devices will be applied to sub-1 nm nodes by 2028 [25], the development of standard cell libraries and automated design platforms based on novel devices can help accelerate design [26] and allow for system-level 2D-based circuit simulation.

**Fig. 4:**
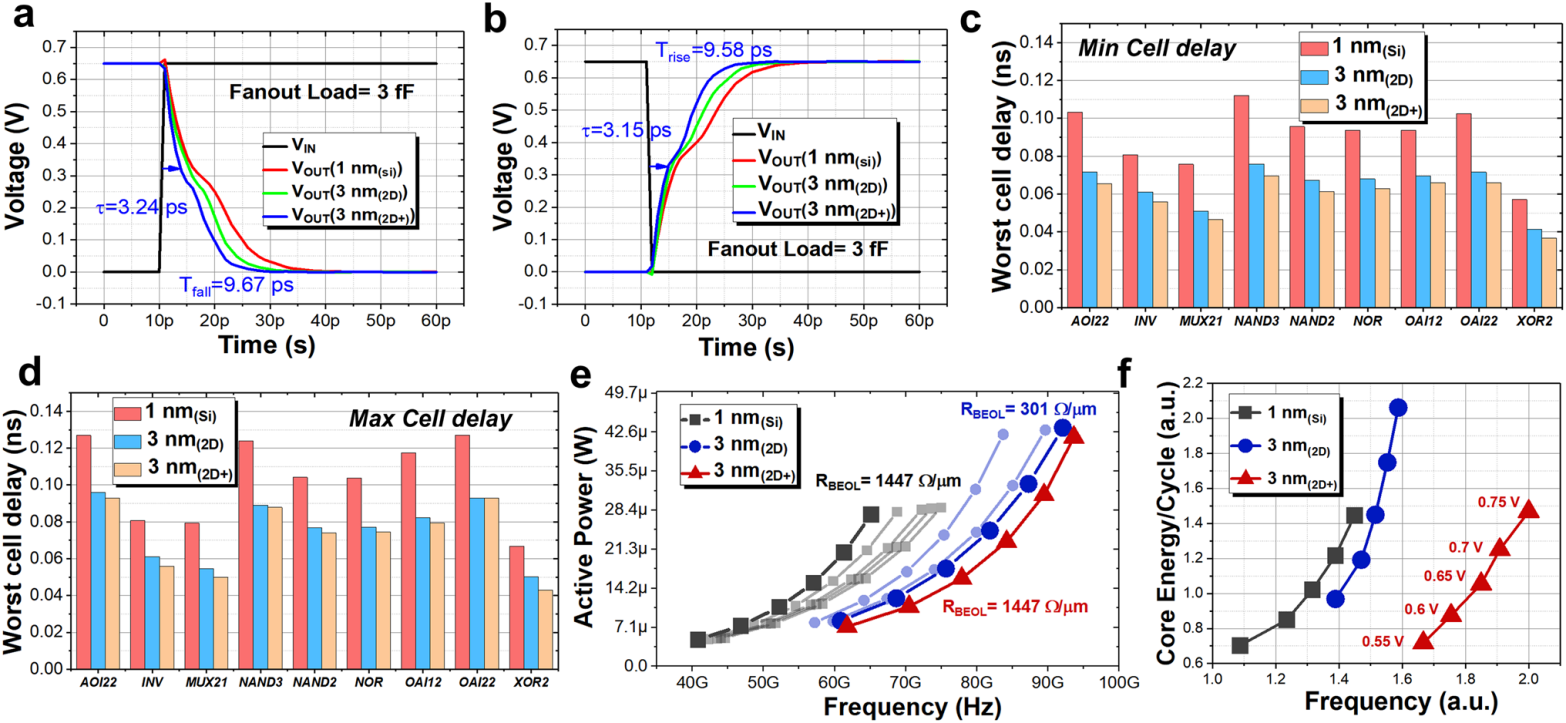
Performance comparison of 16-bit RISC-V CPU system-level circuits based on 2D materials and silicon. **a** and **b** Inverter simulations based on the three devices, including rising and falling edges, from which propagation delays and falling delays can be extracted; **c** and **d** Minimum and maximum cell delays corresponding to 9 different logic gates at *V*_DS_ = 0.65 V. This result highlights the potential of the 2D devices in terms of speed; **e** Active power versus frequency for the RO circuit including R_BEOL_ and *C*_BEOL_. **f** Power versus frequency for the 16-bit RISC-V CPU extracted from the synthesized gate-level netlist, with a trend similar to that of the RO circuit simulation

A system-level evaluation of the Si-based circuits and 2D-based circuits at different nodes is shown in Fig. 4, comparing the performance of these devices in an open-source 16-bit RISC-V CPU in terms of power consumption and latency. After synthesizing the source register transfer-level (RTL) code, the entire CPU consists of 9 combinational logic gates including AOI22, INV, MUX21, NAND3, NAND2, NOR, OAI12, OAI22 and XOR2. As a typical representative of the inverter, we performed gate-level transient simulations. The 3 nm_(2D+)_ node possesses the smallest propagation and descent delays of 3.24 and 9.67 ps, respectively, with a load capacitance of 3 fF (Fig. 4a). The output rising edge demonstrated in Fig. 4b reflects the same trend, with the 1 nm_(Si)_ inverter having the worst dynamic delay.

After standard cell characterization, the 9 logic gates produce different cell delays under different input conditions. Figure 4c, d shows the trends of the minimum cell delay and maximum cell delay, respectively. With operating voltages set to 0.65 V, the results show that all gate delays are significantly reduced for the 3 nm_(2D)_ and 3 nm_(2D+)_ compared to that at 1 nm_(Si)_ node. This can be attributed to the fact that the 2D devices can maintain a high I_ON_/I_OFF_ with such a small *L*_G_ size.

With the introduction of BEOL capacitance and resistance parameters, the RO circuit evaluation provides a more comprehensive and accurate representation of the PPA of the “2D eq 1 nm” scheme. According to IRDS, the resistance and capacitance of BEOL at the 1 nm node are 1447 and 208 aF µm^−1^, respectively. At the same supply voltage, the “2D eq 1 nm” (i.e., 3 nm_(2D+)_) still demonstrates reduced power consumption and speedup over 1 nm_(Si)_ (Fig. 4e). This demonstrates that “2D eq 1 nm,” which takes into account *R*_BEOL_ and *C*_BEOL_, can be an alternative to conventional solutions to achieve cost reductions and efficiencies when the device meets the targeted overall metrics. Notably, although the proposed 2D-based process has the potential to reduce costs by avoiding the complex 3D stacking process and reducing the photomask count, it indeed brings new challenges in terms of yield. One of the main factors affecting yield is the uniformity of 2D material growth. The fabrication process of 2D-NSFETs involves multiple steps, and any small deviation in each step may affect the device performance and ultimately the yield. Moreover, the contact between 2D materials and electrodes also plays an important role in determining the yield. Therefore, the process steps may be largely reduced using the “2D eq 1 nm” scheme, while the product yield of 2D-based devices remains challenging. Compared to 3 nm_(2D)_, “2D eq 1 nm” offers 17% lower power at a fixed frequency and 4% higher frequency at a fixed power. Figure 4e also compares the change in PPA of different node schemes at different *R*_BEOL_. As the *R*_BEOL_ decreases, the operating frequency of RO increases correspondingly.

Figure 4f shows the PPA simulation results of 2D-based 16-bit RISC-V CPU circuit. Such a system-level CPU circuit combines the diversity of various combinational logics and is more in line with the results reflected on the final chip. In the simulation, the supply voltage is gradually increased from 0.8 to 1.2 V in 0.1 V steps. The trend is similar to the one extracted for the 15-step ring oscillator, with a 30% improvement over 3 nm_(2D+)_ and a 48% improvement over 1 nm_(Si)_ for a fixed power.

## Conclusion

This work focuses on the performance evaluation of 2D-NSFETs at advanced nodes and evaluates the PPA of 2D circuits such as RO and CPU system. 2D semiconductors possess excellent lateral size minimization capability and have outstanding performance advantages at advanced nodes. In this work, we propose to replace the next-generation Si-based CFET process at 1 nm node with 2D-NSFET process. The feasibility is verified by cross-scale simulation and system-level circuit simulation. In the future, it can be experimentally verified by building a short-channel 2D-NSFET, which can calibrate the simulation parameters more accurately and thus make the prediction more convincing. Experimentally achieving the desired level of mobility, contact and size of 2D transistors simultaneously is a very important challenge. To realize the high-level integration ability in reality, one of the main limitations is the current challenges in the large-scale synthesis and transfer of high-quality 2D materials. Another limitation lies in the complexity of the fabrication process. The compatibility of 2D materials with existing semiconductor manufacturing processes also needs to be further improved. What’s more, the contact and interface engineering also largely influence the characteristics of 2D-based devices. Although achieving the desired levels of mobility, contact and size simultaneously is indeed a significant challenge in the fabrication of 2D transistors, there have been continuous efforts and advancements in the research community. For example, 2D-based ring oscillator operating at GHz [27], 2D transistors with sub-1 nm gate length [15], semi-metallic antimony contact with *R*_C_ down to 42 Ω µm [28], 2D logic circuits with pW power consumption [29] and high-level mobility of MoS_2_ over 900 cm^2^/(V-s) [30] all have been demonstrated in recent years. The theoretical advantages of 2D-NSFETs in advanced nodes, as demonstrated in this work, still make them an important candidate for next-generation semiconductors.

## Method

In the traditional Si-based NSFET simulation, the device physical models set up include: (1) Carrier transport model, which adopts Schrödinger’s modified drift–diffusion model, i.e., density gradient model, and thin-layer model were used to couple quantum effects; (2) carrier recombination models, including Shockley–Read–Hall (SRH) recombination model, Auger recombination model, etc.; (3) mobility degradation models, including the Philips unified mobility model, Enormal model, high-field saturation model; (4) Fermi statistical models for carrier concentration, which are used in the modeling of the density gradient of the carriers. The case of relatively large degree can get more accurate results; and (5) stress model, which enhances the carrier mobility of the channel. These models were adjusted and calibrated to accurately represent the behavior of Si-NSFETs. Basic materials parameters, such as carrier effective mass, bandgap and dielectric constant, are set according to DFT calculation. The calibrated parameters include carrier mobility, doping concentration and gate oxide thickness within a reasonable range based on the known characteristics of Si-NSFETs.

In 2D semiconductors, in addition to the parameters such as band gap and effective mass set in the previous section, there are also multi-valley parameters. For example, in MoS_2_, the two neighboring energy valleys consist of a *K*-valley and a *Q*-valley. In the single-layer MoS_2_ band, the *Q*-valley has not yet split, so there is only one conduction band bottom. In two-layer MoS_2_, the *Q*-valley splits into two energy bands, and there are two conduction band bottoms. Therefore, the density of states corresponding to the *K* and *Q* energy valleys can be expressed as follows:

$$ \begin{array}{*{20}c} {D_{k} = \frac{{g_{s} g_{Vk} m_{k} }}{{2\pi \hbar^{2} }}U\left( {E - E_{k} } \right)} \\ \end{array} $$

$$ \begin{array}{*{20}c} {D_{Q} = \frac{{g_{s} g_{VQ} m_{Q} }}{{2\pi \hbar^{2} }}\mathop \sum \limits_{i} U\left( {E - E_{Qi} } \right)} \\ \end{array} $$

where U is a step function. Since theoretically WS_2_ has relatively high mobility and I_ON_ in the 2D-FETs, this paper has conducted energy band calculations for single-layer WS_2_ materials by using the DFT method, in which the exchange correlation generalization is based on the PBE function of GGA. In technology computer-aided design, the multi-valley model and 2D density of states model are set up to simulate 2D-FETs more accurately.

For the RO circuit simulation, we first perform technology computer-aided design simulations for 2D-NSFET devices and Si-NSFET devices, respectively. In the former, we input the corresponding physical model and material parameters to obtain the basic electrical data of the devices. After obtaining the C-V and I-V data based on the device simulation, the data are substituted into the BSIM-CMG model for parameter fitting based on the MBP platform to ensure that the RMS error is reduced to a certain level (RMS < 3%), and then, the RO circuit is constructed for the circuit simulation to extract the circuit PPA characteristics, as shown in Fig. S10. For the simulation of Si-based transistors at 1 nm node, complementary structure is used. Despite some differences in models and parameters, researchers currently borrow BSIM models for circuit simulation, e.g., the BSIM-IMG model can be used for dual-gate WS_2_ transistors [16], the BSIM-CMG model can be used for planar MoS_2_ transistors [31], and so on. For 2D-NSFET structure used in this work, the BSIM-CMG model is proposed to be used. Figure S11 gives the calibration curve for the 3 nm_(2D+)_ node.

Regarding the establishment of the system-level simulation platform, the SPICE netlist is written based on the model and the back-end interconnect parasitic parameters are ignored. Then, we perform the standard cell characterization to extracts the timing and power consumption information inside the transistor. After building a standard cell library file, the library file is then converted into a non-text.db file using the Library Compiler tool. At the same time, the RTL code describing the open-source 16-bit RISC-V CPU is input, and logic synthesis is performed based on the Design Compiler tool. Finally, the gate-level netlist is obtained to output the power consumption, delay and other information of the gate-level circuit system, as shown in Fig. S12.

First, Si/SiO_2_ was selected as the sample substrate and the substrate is patterned to form trenches with a depth of 40 nm by etching. Subsequently, electron beam evaporation (EBE) was used to deposit gold (Au) layer on the substrate with a thickness of 40 nm. Next, 15-nm-thick layers of hafnium oxide (HfO_2_) are uniformly deposited on the surface of the sample by atomic layer deposition (ALD) technique. On this basis, wafer-scale large-size three-layer molybdenum disulfide (MoS_2_) thin films were transferred to the sample surface. The surface of the MoS_2_ film was patterned, and electron beam evaporation was again used to deposit titanium/gold (Ti/Au) as the source–drain electrodes, with a thickness of 5 nm for Ti and 40 nm for Au. Next, the MoS_2_ films were patterned and etched using inductively coupled plasma (ICP) etching to define the channel regions. A 15-nm-thick HfO_2_ layer was deposited again by ALD, followed by a 60-nm-thick gold layer by electron beam evaporation, and a 15-nm-thick HfO_2_ layer was deposited again by ALD. Both deposited HfO_2_ layers were used as a gate oxide layer. Then, the MoS_2_ film was transferred again, and Ti/Au deposition of the source–drain electrodes as well as the formation of the channel region was realized by a similar deposition and etching process. After these steps, a 15-nm-thick HfO_2_ layer was deposited by ALD. The sample is patterned again, and a 110-nm-thick gold layer is deposited by electron beam evaporation to form the top gate electrode. Finally, the sample was patterned again and the HfO_2_ layer was etched to form a via hole. A 110-nm-thick gold layer was deposited in the via holes to interconnect the source–drain and gate electrodes of each MoS_2_ field-effect transistor layer, respectively. Throughout the preparation process, all lithographic patterning treatments were performed using laser direct writing (MicroWriter ML3). The MoS_2_ film was synthesized by dual-temperature CVD method. The transfer of MoS_2_ was achieved using a two-layer MMA/PMMA coating technique with heating, mechanical stripping, stacking and annealing steps.

## Supplementary Information

Below is the link to the electronic supplementary material.

Supplementary file 1 (DOCX 9784 KB)
