# Correction: A Valuable and Low-Budget Process Scheme of Equivalized 1 nm Technology Node Based on 2D Materials

**DOI:** 10.1007/s40820-025-01747-8

**Published:** 2025-06-12

**Authors:** Yang Shen, Zhejia Zhang, Zhujun Yao, Mengge Jin, Jintian Gao, Yuhan Zhao, Wenzhong Bao, Yabin Sun, He Tian

**Affiliations:** 1 College of Integrated Circuit Science and Engineering, Shanghai Key Laboratory of Multidimensional Information Processing, East China Normal University, Shanghai, 200241 People’s Republic of China; 2 Institute of Microelectronics and Beijing National Research Center for Information Science and Technology (BNRist), Tsinghua University, Beijing, 100084 People’s Republic of China; 3 State Key Laboratory of ASIC and System, School of Microelectronics, Fudan University, Shanghai, 200433 People’s Republic of China; 4 Shaoxin Laboratory, Shaoxing, 312000 People’s Republic of China

**Correction to: Nano-Micro Letters (2025) 17:191** 10.1007/s40820-025-01702-7

Following the publication of the original article [1], the authors reported an error in Fig. 3(b), and the figure legend was reversed.

The correct Fig. 3 has been provided in this orrection.

The incorrect Fig. 3 is:

**Figure:**
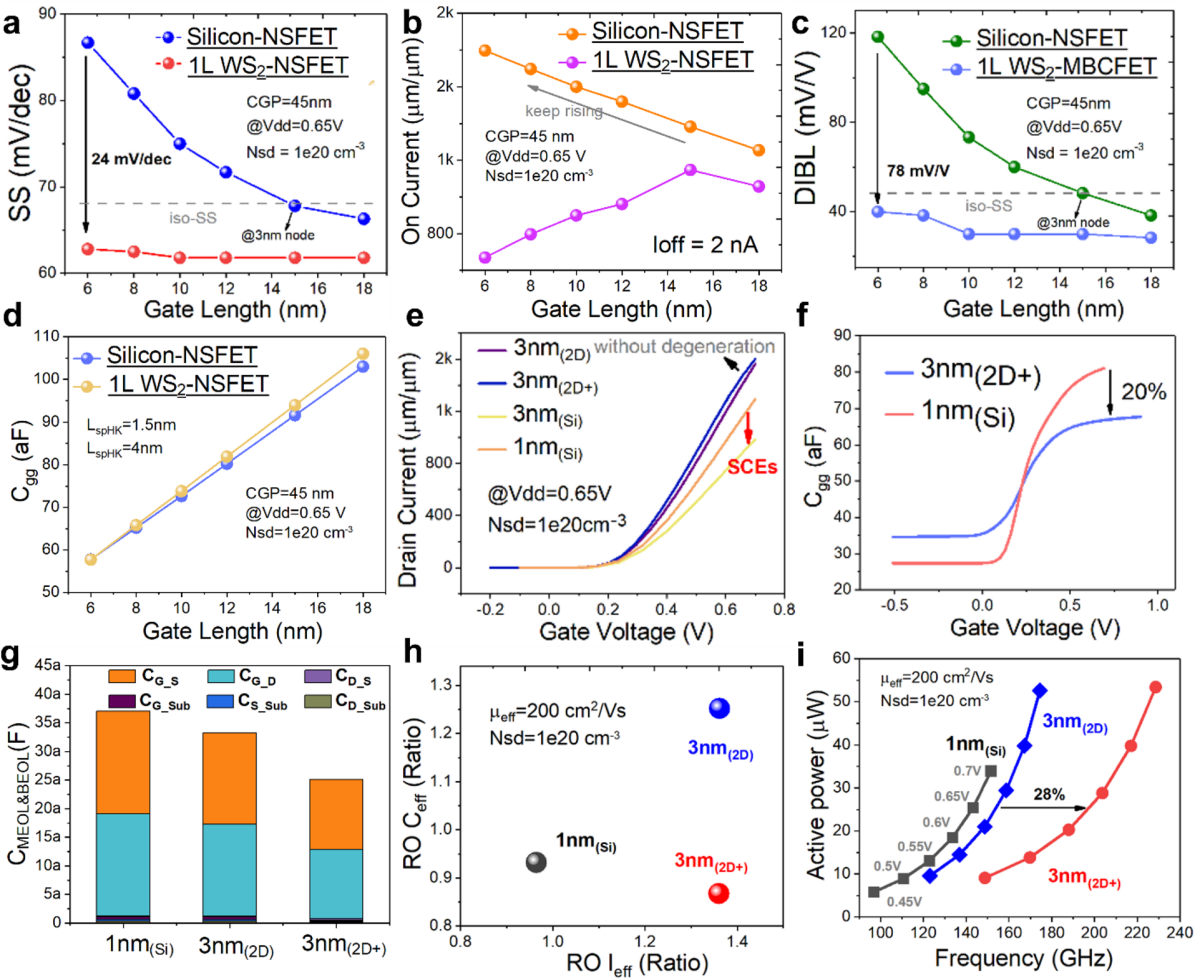
**Fig. 3**. Comparison of electrical characteristics of devices at 3 nm_(2D+)_ node, 3 nm_(2D)_ node and 1 nm_(Si)_ node, with fixed CGP of 45 nm and *L*_G_ shrinking from 18 to 6 nm for Si-NSFETs and WS_2_ NSFETs. **a** SS variation, the SS of Si-NSFETs degrades drastically when the L_G_ is smaller than 12 nm, and **b** *I*_ON_ variation, thanks to the smaller feature length of 2D materials, the *I*_ON_ of WS_2_ NSFETs continues to increase even with the *L*_G_ scaled to 6 nm, while the I_ON_ of Si-NSFETs degrades continuously when the *L*_G_ is reduced to 6 nm; **c** DIBL variation, which follows a similar trend to that of SS; **d** C_gg_ variations, with EOT and gate size being the main influences on *C*_gg_; **e** Linear transfer characteristics corresponding to four devices, at 3 nm_(2D)_, 3 nm_(2D+)_, 3 nm_(Si)_ and 1 nm_(Si)_; **f** *C*_gg_-*V*_GS_ relationship for 3 nm_(2D+)_ and 1 nm_(Si)_ counterparts, which is reduced by 20% for the 3 nm_(2D+)_ due to shortened *L*_G_; **g** C_MEOL&BEOL_ comparison with middle end of line (MEOL) and back end of line (BEOL) parasitic capacitances; **h** Comparison of equivalent capacitances and equivalent currents extracted from RO circuits; and **i** Power–frequency comparison of RO circuits

The correct Fig. 3 is:

**Figure:**
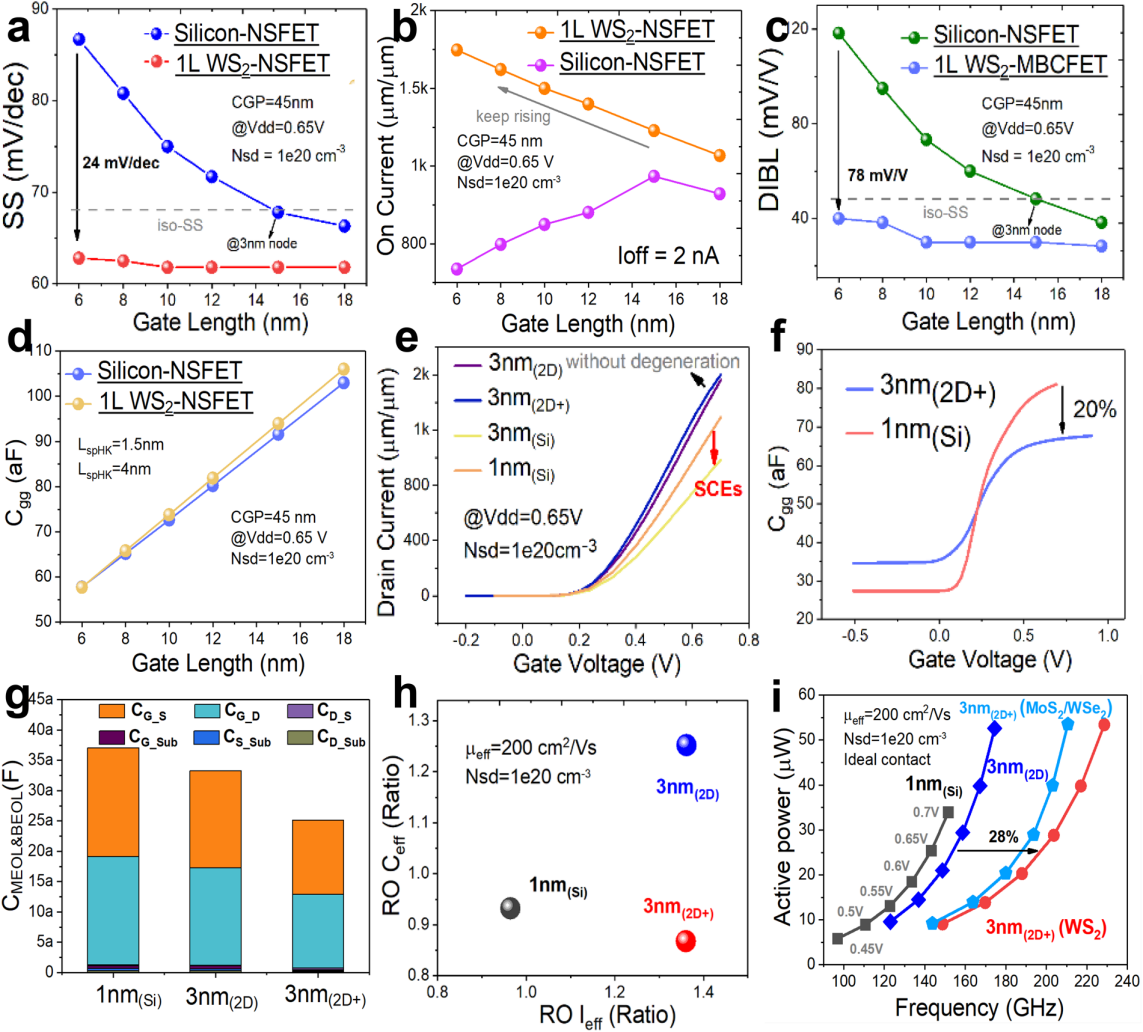
**Fig. 3**. Comparison of electrical characteristics of devices at 3 nm_(2D+)_ node, 3 nm_(2D)_ node and 1 nm_(Si)_ node, with fixed CGP of 45 nm and *L*_G_ shrinking from 18 to 6 nm for Si-NSFETs and WS_2_ NSFETs. **a** SS variation, the SS of Si-NSFETs degrades drastically when the L_G_ is smaller than 12 nm, and **b** *I*_ON_ variation, thanks to the smaller feature length of 2D materials, the *I*_ON_ of WS_2_ NSFETs continues to increase even with the *L*_G_ scaled to 6 nm, while the I_ON_ of Si-NSFETs degrades continuously when the *L*_G_ is reduced to 6 nm; **c** DIBL variation, which follows a similar trend to that of SS; **d** C_gg_ variations, with EOT and gate size being the main influences on *C*_gg_; **e** Linear transfer characteristics corresponding to four devices, at 3 nm_(2D)_, 3 nm_(2D+)_, 3 nm_(Si)_ and 1 nm_(Si)_; **f** *C*_gg_-*V*_GS_ relationship for 3 nm_(2D+)_ and 1 nm_(Si)_ counterparts, which is reduced by 20% for the 3 nm_(2D+)_ due to shortened *L*_G_; **g** C_MEOL&BEOL_ comparison with middle end of line (MEOL) and back end of line (BEOL) parasitic capacitances; **h** Comparison of equivalent capacitances and equivalent currents extracted from RO circuits; and **i** Power–frequency comparison of RO circuits

The original article [1] has been corrected.
