# Supplementary material for: A Valuable and Low-Budget Process Scheme of Equivalized 1 nm Technology Node Based on 2D Materials
Source: Nanomicro Lett. 2025 Mar 18;17:191. doi: 10.1007/s40820-025-01702-7 (PMC11920538; doi:10.1007/s40820-025-01702-7)
Supplement: Supplementary file 1 — (DOCX 9784 KB) [file 40820_2025_1702_MOESM1_ESM.docx]

Supporting Information for

**A Valuable and Low-Budget Process Scheme of Equivalized 1 nm Technology Node Based on 2D Materials**

Yang Shen^1^, Zhejia Zhang^3^, Zhujun Yao^2^, Mengge Jin^1^, Jintian Gao^2^, Yuhan Zhao^2^, Wenzhong Bao^3,4^*, Yabin Sun^1^*, He Tian^2^*

^1^College of Integrated Circuit Science and Engineering, Shanghai Key Laboratory of Multidimensional Information Processing, East China Normal University, Shanghai 200241, P. R. China

^2^Institute of Microelectronics and Beijing National Research Center for Information Science and Technology (BNRist), Tsinghua University, Beijing 100084, P. R. China

^3^State Key Laboratory of ASIC and System, School of Microelectronics, Fudan University, Shanghai 200433, P. R. China

^4^Shaoxin Laboratory, Shaoxing 312000, P. R. China

*Corresponding authors. E-mail: [tianhe88@tsinghua.edu.cn](mailto:tianhe88@tsinghua.edu.cn) (He Tian); [ybsun@cee.ecnu.edu.cn](mailto:ybsun@cee.ecnu.edu.cn) (Yabin Sun); [baowz@fudan.edu.cn](mailto:baowz@fudan.edu.cn) (Wenzhong Bao)

**Supplementary Figures**

**Fig. S1** Detailed process flow diagram of the fabricated MoS_2_ NSFET, with two layers of conducting channel. The channel is actually three-layer 2D MoS_2_. The source and drain electrode at different layer are connected finally with each other by etching and metal deposition

**Fig. S2** 5 groups of transfer and output characteristics corresponding to the fabricated MoS_2_ NSFET, which show natural and uniform device behaviors

**Fig. S3** Geometric parameters of Si-CFET and 2D-NSFET at different technology nodes were used in our device simulations, where L_spLK_ is the low-k spacer length and L_spHK_ is the high-k spacer length. R_BEOL_ and C_BEOL_ are interconnect resistance and capacitance at back end of line (BEOL)

**Fig. S4** The constructed Si-CFET at 1 nm node and 2D-NSFET at 3 nm node in our device simulation. FP represents Fin pitch and CGP represents contacted gate pitch. The Si-CFET consists of p-type Si-NSFET stacking on n-type Si-NSFET

**Fig. S5** The cross-scale simulation framework to investigate device behavior. Underlay material parameters were obtained from DFT calculation, and then the parameters were transferred into device simulation. After calibrated electric performance to experimental results, the whole model can be used to predict

**Fig. S6** DFT calculated material parameter of 3 layer MoS_2_ for experimental data calibration and WS_2_ for prediction. The parameter mobility is set for calibration or target case

**Fig. S7** **a** The calculated band structure of monolayer WS_2_, with K valley and Q valley labeled. **b** The density of states distribution of 1D, 2D, 3D, and Graphene materials, among which the constant function is adopted to the 2D semiconductors for studying 2D-FETs more accurately

**Fig. S8** The plotted best gate length, mobility, I_ON_, V_dd_ and leakage in previous experimental reports, i.e., experimental case. And the target case presents the expectant parameters and performances. With target mobility of 200 cm^2^/V-s and a source-drain doping concentration of 1e20 cm^-3^, the I_ON_ of NMOS and PMOS corresponding to the 3 nm_(2D+)_ can reach 1.291 mA/um and 1.382 mA/um

**Fig. S9** The extracted C_D_S_, C_G_sub_, C_D_sub_ and C_S_sub_ at different technology nodes. As the sizes of typical devices change, C_D_S_ increase with shortening of channel length, while the other 3 parts related to substrate decrease

**Fig. S10** The simulation framework to obtain Si-based and 2D-based circuit performance. After introducing Si parameter and 2D parameter, device simulation output C-V and I-V and SPICE model can be calibrated. If final PPA simulation could not meet our demand, the requirements for device, i.e., target device parameters, should be enhanced

**Fig. S11** The calibration of BSIM-CMG model to simulated device characteristics at 3 nm_(2D+)_ node. The root mean square (RMS) of all calibrations are controlled below 3%

**Fig. S12** System-level benchmark framework, based on 16-bit RISC-V CPU. The calibrated SPICE model above is input of module “Timing/Power Characterization”, from which timing/power information of standard cell can be obtained
